# VARIABILITY IN ACCESS TO VA’S AID AND ATTENDANCE PENSION BENEFIT: A MIXED-METHODS STUDY

**DOI:** 10.1093/geroni/igz038.2727

**Published:** 2019-11-08

**Authors:** Kali S Thomas, Emily Corneau, Stefanie Gidmark, Taylor Rickard, Susan Allen

**Affiliations:** 1 Providence VA Medical Center, Providence, Rhode Island, United States; 2 Brown School of Public Health, Providence, Rhode Island, United States

## Abstract

The Veterans Benefit Administration’s (VBA) Aid and Attendance enhanced pension benefit (A&A) is available to older, low-income Veterans who require assistance meeting their daily needs. However, reports indicate that A&A is underutilized with only 1/3 of eligible Veterans receiving this benefit. The objective of this mixed methods study is to characterize the variability in A&A enrollment across VA Medical Centers (VAMCs) and determine factors attributable to the variation. Using VA administrative data, we calculated the rate of enrollment in A&A among Veterans receiving pension. We then purposefully sampled 16 Chiefs of Social Work at VAMCs with the highest (n=7) and lowest (n=9) rates of enrollment. Interviews were transcribed, coded, and analyzed using conventional qualitative research methods. The rate of enrollment in A&A varies from <1% to 23% across VAMCs. VAMCs that had higher rates of enrollment were larger and more likely to be located in the South and Mid-Atlantic regions. Respondents at sites with low rates of enrollment indicate that education around the eligibility criteria is needed for VAMC staff. They also report that outreach to Veterans about this benefit is limited. Respondents at VAMCs with high rates of enrollment indicate that the relationships with VBA and Veterans Service Organizations facilitates access. Universally, respondents viewed the A&A benefit positively and note that it helps meet Veterans’ long-term care needs. As the Veteran population continues to age, it is important that VA ensure equal access to A&A for eligible Veterans. Implications of these findings and next steps will be discussed.

